# SEOM clinical guideline on heritable TP53-related cancer syndrome (2022)

**DOI:** 10.1007/s12094-023-03202-9

**Published:** 2023-05-03

**Authors:** Ana Beatriz Sánchez-Heras, Teresa Ramon y Cajal, Marta Pineda, Elena Aguirre, Begoña Graña, Isabel Chirivella, Judit Balmaña, Joan Brunet, the SEOM Hereditary Cancer Working Group and AEGH Hereditary Cancer Committee

**Affiliations:** 1 Medical Oncology Department, Hospital General Universitario de Elche, Elche, Alicante, Spain; 2 Medical Oncology Service, Hospital Sant Pau, Barcelona, Spain; 3 Hereditary Cancer Program, Catalan Institute of Oncology, Institut d’Investigació Biomèdica de Bellvitge (IDIBELL), ONCOBELL Program, L’Hospitalet de Llobregat, Barcelona, Spain; 4 Consortium for Biomedical Research in Cancer, CIBERONC, Carlos III Institute of Health, Madrid, Spain; 5 Medical Oncology Department, Hospital Quironsalud, Zaragoza, Spain; 6 Medical Oncology Department, University Hospital A Coruña, 15006 A Coruña, Spain; 7 Medical Oncology Department, INCLIVA Biomedical Research Institute, University of Valencia, Valencia, Spain; 8 Medical Oncology Department, Hospital Vall d’Hebron, and Hereditary Cancer Genetics Group, Vall d’Hebron Institute of Oncology, Barcelona, Spain; 9 Medical Oncology Department, Catalan Institute of Oncology, University Hospital Josep Trueta, University of Girona, Girona, Spain; 10 Hereditary Cancer Program, Catalan Institute of Oncology, Girona Biomedical Research Instiute (IDIBGI), Girona, Spain

**Keywords:** Li-Fraumeni syndrome, *TP53*, Cancer, Pathogenic variants

## Abstract

Li-Fraumeni syndrome is caused by heterozygous germline pathogenic variants in the *TP53* gene. It involves a high risk of a variety of malignant tumors in childhood and adulthood, the main ones being premenopausal breast cancer, soft tissue sarcomas and osteosarcomas, central nervous system tumors, and adrenocortical carcinomas. The variability of the associated clinical manifestations, which do not always fit the classic criteria of Li-Fraumeni syndrome, has led the concept of SLF to extend to a more overarching cancer predisposition syndrome, termed hereditable *TP53-*related cancer syndrome (hTP53rc). However, prospective studies are needed to assess genotype–phenotype characteristics, as well as to evaluate and validate risk-adjusted recommendations. This guideline aims to establish the basis for interpreting pathogenic variants in the *TP53* gene and provide recommendations for effective screening and prevention of associated cancers in carrier individuals.

## Introduction

Li-Fraumeni syndrome (LFS) is characterized by a high risk of developing a wide variety of malignant tumors in childhood and adulthood, caused by heterozygous germline pathogenic variants in the *TP53* gene. Based on penetrance data from familial presentation cases, the highest cumulative incidences in females are 56% and 15% for breast cancer (BC) and soft tissue sarcoma (STS) respectively, while in males, they said incidences are 20% both for STS and for brain cancer [1]. The predominant cancers in people with LFS are osteosarcoma and STS, premenopausal BC, brain tumors, and adrenocortical carcinoma (ACC); similarly, rare tumors, such as choroid plexus carcinoma, hypodiploid acute lymphoblastic leukemia, anaplastic embryonal rhabdomyosarcoma, subtype sonic hedgehog-driven medulloblastoma, and jaw osteosarcoma are highly suggestive of LFS [2, 3].

In recent years, the development of multigene panels for cancer has resulted in increased germline *TP53* testing in oncology patients. Therefore, more tumors potentially linked to germline alterations of *TP53* have been reported. The heterogeneity of clinical presentations associated with germline *TP53* alterations justifies the extending the LFS concept to a wider cancer predisposition syndrome designated heritable *TP53*-related cancer (hTP53rc) syndrome. Moreover, cancer risk and cancer surveillance recommendations are evolving as new genotype–phenotype relationships are being described. Here, we aim to provide an updated clinical guideline for the identification of *TP53* germline pathogenic variants, cancer risk estimation, and surveillance recommendations.

### TP53 gene testing

Criteria for germline *TP53* variant testing have evolved since Birch´s first definition in 1994. Patients with cancer who meet the latest modified ´Chompret Criteria´ should be tested for germline *TP53* variants (Table 1). Likewise, individuals should be tested who develop a second primary tumor within the radiotherapy field of a first core *TP53*-tumor that occurred before 46 years. Cascade genetic testing of the germline disease-causing *TP53* variant should be offered to adult family members.

**Table 1:** Criteria for germline *TP53* testing (modified from Frebourg et al. EJHG 2020)

Adults	Cancer patients	1. All patients who meet the modified ‘Chompret Criteria’: Familial presentation: proband with a TP53 core tumor (breast cancer, soft-tissue sarcoma, osteosarcoma, central nervous system tumor, adrenocortical carcinoma) before **46** years AND at least one first- or second-degree relative with a core tumor before **56** years (except breast cancer if the proband has breast cancer); or Multiple primitive tumors: proband with multiple tumors, including two TP53 core tumors, the first of which occurred before **46** years, irrespective of family history; or Rare tumors: patient with adrenocortical carcinoma, choroid plexus carcinoma, or anaplastic embryonal rhabdomyosarcoma, irrespective of family history, or Very early-onset breast cancer: Breast cancer before **31** years, irrespective of family history 2. Patients who develop a second primary tumor within the radiotherapy field of a first core *TP53* tumor which occurred before 46 years
	Pre-symptomatic testing	3. All first-degree relatives of individuals with germline disease-causing *TP53* variants
Children and adolescents	Cancer patients	4. Patients meeting 1 or 2 criteria above or all patients presenting with hypodiploid acute lymphoblastic leukemia (ALL); or unexplained sonic hedgehog-driven medulloblastoma, or jaw osteosarcoma
	Pre symptomatic testing	5. All first-degree relatives of individuals with a germline disease-causing *TP53* variant, if the variant confers a high cancer risk in childhood: Childhood cancers have been observed in the family or This variant has already been detected in other families with childhood cancers or It is a dominant-negative missense variant or If there is insufficient evidence in the databases or registries to determine the childhood cancer risk

Child and adolescent cancer patients should also be tested for germline *TP53* variants if presenting with hypodiploid acute lymphoblastic leukemia (ALL), unexplained sonic hedgehog-driven medulloblastoma, or osteosarcoma of the jaw. Healthy children who are first-degree relatives of individuals with a germline disease-causing *TP53* variant should be offered predictive testing if the genetic variant confers a high cancer risk in childhood (reported in families with childhood cancers carrying the same pathogenic variant, childhood cancers have been observed within the family, or it is a dominant-negative missense variant) [4]. When there is insufficient evidence to determine the childhood cancer risk, the decision to perform genetic testing in children will be made on a case-by-case basis.

### Interpretation of TP53 variants

Constitutional *TP53* variants are classified as per *TP53*-specific ACMG/AMP guidelines, which are based on the integration of multiple lines of evidence, including variant frequency in general population, information on phenotype, bioinformatic predictions, and functional data [5]. Unlike loss-of-function variants (nonsense, frameshift, splicing, gross rearrangements), the interpretation of the most common *TP53* missense variants is not always obvious. Furthermore, a subclass of missense variants exert a dominant-negative effect resulting in mutant proteins that form tetramers with wild-type *TP53*, inhibiting the transcriptional activity of the protein complex and causing larger defects in response to DNA damage [6–10].

The penetrance of *TP53* variants is incomplete and varies depending on the type of variant and modifying factors [4]. Dominant-negative missense *TP53* variants have been reported generally as highly penetrant and detected in families with childhood cancers [11]. In contrast, debate continues regarding phenotype-genotype correlation associated with loss of function and non-dominant negative variants [1, 11, 12]. Also, the different penetrance among carriers from the same family suggests the coexistence of genetic and environmental modifiers [13, 14].

**Fig. 1:**
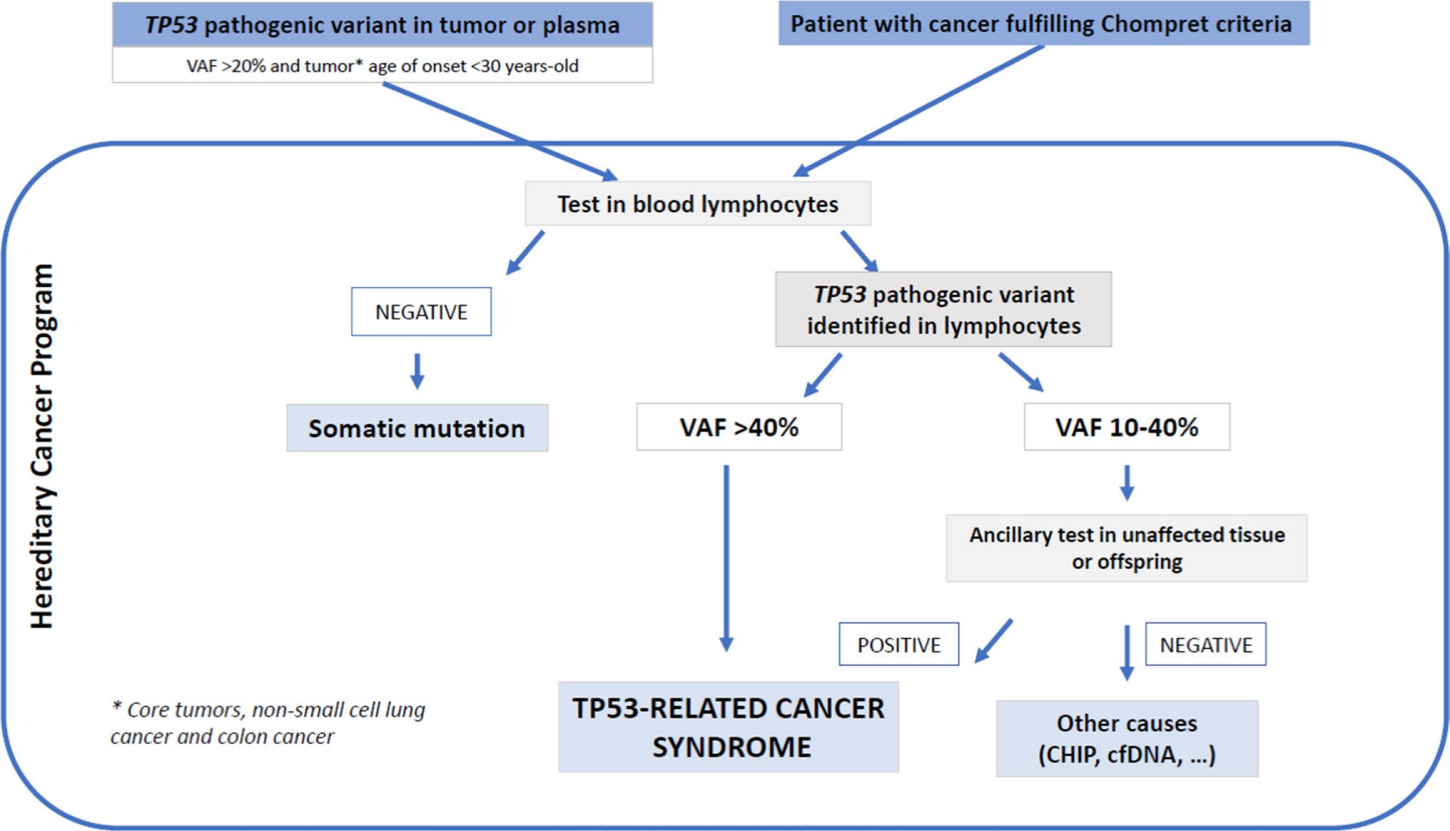
Algorithm proposal for the interpretation of *TP53* pathogenic variants

The detection of a *TP53* pathogenic variant in the tumor or plasma of patients with early onset diagnosis (< 30 years of age) of *TP53* core tumors (breast cancer, soft-tissue sarcoma, osteosarcoma, central nervous system tumor, adrenocortical carcinoma), or other tumors exhibiting an enriched germline conversion rate, such as non-small cell lung cancer or colon cancer, requires further assessment at a Hereditary Cancer Unit (Fig. 1) [15]. In individuals who meet Chompret’s criteria, the identification of a *TP53* (likely) pathogenic variant with a variant allele frequency (VAF) of 40–50% in lymphocyte blood testing can be presumed to be germline and associated with a *TP53*-related cancer syndrome. Additionally, the detection of a *TP53* variant at lower VAFs (10–40%) in blood DNA (suggesting germline mosaicism) should be confirmed with supplementary tests that assess the presence of the *TP53* variant in unaffected tissues with no lymphocyte content (e.g., skin biopsy, follicle bulbs), to rule out other possibilities, such as *TP53* clonal hematopoiesis of indeterminate potential (CHIP) or circulating tumor DNA [16–18] (Fig. 1). For interpretation purposes, offspring testing might be also useful and informative after identifying additional carriers.

When a *TP53* gene (likely) pathogenic variant is found in the setting of multigene panel testing in a patient not fulfilling Chompret criteria, causes other than those of germline origin must be ruled out and results should be discussed among a multidisciplinary board.

### Surveillance and management recommendations

#### Surveillance

Previous debate about the possible lack of benefit of clinical surveillance in the heritable* TP53-*related cancer syndrome has been resolved after reporting the clinical impact of the Toronto protocol on patient outcomes. This protocol has demonstrated improved 5-year survival rates among individuals undergoing surveillance compared to the non-surveillance group (88% vs 59.6%), with reported psychological benefits [19–22]. Patient surveillance also proved to be cost-effective [20, 23].

In 2017, an international consortium established a list of cancer screening recommendations for individuals with germline disease-causing *TP53* variants [24]. More recently, two additional consensus surveillance guidelines have been published, the first by the United Kingdom Cancer Genetics Group (UKCGG) Consensus Group [25] and the second by the European Reference Network (ERN) on Genetic Tumour Risk Syndromes (GENTURIS) [4].

Female breast cancer is the most frequent LFS-associated cancer [26], especially in premenopausal women [2, 11, 27]. Breast magnetic resonance imaging (MRI) has been demonstrated to be more sensitive than mammography in women with familial and genetic predisposition, including *TP53* carriers, in first and subsequent rounds for detection of early breast cancer [28, 29]. Annual breast MRI can be alternated with whole-body MRI (WBMRI) at 6 months. Mammography is not recommended so as to avoid radiation [4, 24].

In *TP53* carriers there is high lifetime risk of developing a sarcoma, with osteosarcomas usually diagnosed in children [2, 11]. Annual WBMRI with diffusion-weighted imaging, vertex to feet, using fast sequences, without gadolinium contrast is recommended. This procedure is effective for the early detection of solid tumors in addition to sarcomas. A meta-analysis of baseline WBMRI in *TP53* variant carriers yielded an overall estimated detection rate of new tumors of 7%, with a false-positive rate of 42.5% [30]. Absence of clinical correlation and targeted imaging will help to prevent unnecessary biopsies. Radiologists should follow the guidelines for WBMRI acquisition, interpretation, and reporting for cancer screening [31]. Another important issue is the need for sedation to perform WBMRI in children, with the risk that this entails [30, 32, 33]. Most tumors are detected in early stages and are curable with surgery. With decreased therapeutic intensity, comes decreased therapy-associated complications and can lead to improved quality of life [30].

The risk of brain tumors lasts a lifetime [2, 11]. In various studies, baseline brain MRI has a sensitivity of 60% and specificity of 80%. The baseline cancer detection rate ranges from 1.7% [34] to 8.6% [35]; the cumulative cancer detection rate is 13.6% [20]. The recommendation is for yearly brain MRIs (first, with gadolinium-based contrast and, if normal, subsequent MRIs may be done without contrast).

The risk of ACC in children is approximately 4% and decreases after the first decade of life. Surveillance consisting of clinical examination for signs of virilization, early puberty, Cushing-like features, determination of 17-OH-progesterone, total testosterone, dehydroepiandrosterone sulfate and androstenedione, while abdominal and pelvic ultrasound enables early diagnosis to be made, mostly in stage I, with better chances of cure and survival [20, 36].

For hematopoietic malignancies, there is no evidence that screening procedures lead to a presymptomatic diagnosis and improved survival in healthy individuals [37]. In oncology patients who have received leukemogenic drugs (alkylating agents, topoisomerase inhibitors), annual complete blood count is recommended [4, 24, 38].

If the *TP53* carrier patient has received abdominal radiotherapy for the treatment of a previous cancer or if there is a familial history of colorectal tumor suggestive of increased risk, colonoscopy is recommended every five years from 18 years of age onward.

Of note, the spectrum of tumors depends based on the age of the proband; consequently, screening measures should be adapted throughout life.

#### Who should be offered surveillance?

The full screening protocol should be offered to patients harboring *TP53* likely pathogenic or pathogenic variant, whether germline or constitutional mosaicism. As previously mentioned for genetic screening, we emphasize that surveillance measures in children should only be offered when the variant confers a high cancer risk in childhood. Additionally, those patients affected with cancer satisfying classic LFS criteria without a pathogenic *TP53* variant identified should be offered surveillance [24, 25].

#### When to begin surveillance?

In general, it is recommended that surveillance commence as soon as the carrier status is known and be maintained throughout life (Table 2). Whenever possible, screening should continue even after the diagnosis of a primary malignant tumor, adapted to the disease stage or situation. Said surveillance is integrated into the specific clinical follow-up of the cancer diagnosed.

**Table 2:** Surveillance recommendations for *TP53* pathogenic variant carriers

Children (birth to age 18 years)
Whole physical exam with blood pressure, weight, and height, with special attention to signs of virilization, early puberty, Cushing-like features, and focal neurologic deficit. Every 4–6 months
Abdominal and pelvic ultrasound. Every 3–6 months
Total testosterone, dehydroepiandrosterone sulfate, and androstenedione. Every 3–6 months when abdominal ultrasound does not allow proper imaging of the adrenal glands
Brain MRI (first MRI with gadolinium enhancement and, if normal, thereafter without contrast). Annual
Whole-body MRI without gadolinium enhancement. Annually
Complete blood count^a^. Annually
Adults (> 18 years)
Whole physical exam. Every 6 months
Brain MRI (first MRI with gadolinium enhancement and, if normal, thereafter, without contrast). Annually
Whole-body MRI without gadolinium contrast. Annually
Colonoscopy. Every 5 years^b^
Complete blood count^a^. Annually
Women:
Clinical breast exam, starting at age 20. Every 6 months
Breast MRI (ages 20–75). Annually
Recommend risk-reducing bilateral mastectomy

^a^If previous leukemogenic drugs

^b^If abdominal radiotherapy or family history of colorectal cancer

#### Who should coordinate screening?

Screening in children should be managed by a pediatric oncologist or a trained specialist. In the case of adults, it should preferably be coordinated by a specialist trained in genetics and knowledgeable about the syndrome. For healthy carriers, this high-risk surveillance program should be performed in a non-oncological setting. Ideally, patients should be referred to specialized units and screening findings be discussed in specific multidisciplinary boards.

Individuals harboring a germline pathogenic variant should be encouraged to lead a healthy lifestyle by avoiding smoking, exposure to known carcinogens, and sunlight, as well as by using high protection factor sunscreen, eating a healthy diet, and exercising.

### Management

Typically, LFS tumors are treated according to standard protocols, except for surgical treatment for breast cancer. Bilateral mastectomy is recommended instead of breast-conserving surgery to avoid the need for radiotherapy and reduce the risk of a second primary breast cancer [27].

There is clinical and in vivo evidence of increased sensitivity to ionizing radiation and genotoxic chemotherapies [39, 40]. However, until more evidence is gathered, standard chemotherapy regimens should be administered. Ideally, treatment should be personalized using non-leukemogenic drugs.

Despite recent studies demonstrating that the risk of radio-induced cancers is lower than previously reported [41, 42], exposure to radiation should be avoided whenever possible [43].

Risk-reducing bilateral mastectomy should be discussed with female healthy carriers and breast cancer patients [4, 24, 25].

### Future directions

Different consensus groups advise against a surveillance protocol based on genotype and modifiers, inasmuch as they have not been validated prospectively [24, 25]. Clinical and genetic registries are necessary to assess genotype–phenotype features. The reported outcomes of surveillance of *TP53* pathogenic variant carriers will enable us to evaluate and validate screening recommendations that might be tailored on the type of variant, genetic modifiers, and family history.

In the meantime, expert multidisciplinary boards should be set up for consultation of clinical interpretation of *TP53* variants and to establish the best surveillance recommendations for the individual and the family. We propose the creation of referral centers for individuals with heritable *TP53*-related cancer syndrome.
